# Genetic and antiretroviral drug resistance mutations analysis of *reverse transcriptase and protease* gene from Pakistani people living with HIV-1

**DOI:** 10.1371/journal.pone.0290425

**Published:** 2023-08-24

**Authors:** Dilsha Siddiqui, Uzma Badar, Mahnoor Javaid, Nida Farooqui, Sharaf Ali Shah, Ayesha Iftikhar, Faisal Sultan, Fatima Mir, Sofia Furqan, Syed Faisal Mahmood, Syed Hani Abidi

**Affiliations:** 1 Department of Biological and Biomedical Sciences, Aga Khan University, Karachi, Pakistan; 2 Department of Genetics, University of Karachi, Karachi, Pakistan; 3 Medical College, Aga Khan University, Karachi, Pakistan; 4 Bridge Consultants Foundation, Karachi, Pakistan; 5 Shaukat Khanum Memorial Hospital and Research Centre, Lahore, Pakistan; 6 Department of Pediatrics and Child Health, Aga Khan University, Karachi, Pakistan; 7 National AIDS Control Program, Ministry of Health, Islamabad, Pakistan; 8 Department of Medicine, Aga Khan University, Karachi, Pakistan; 9 Department of Biomedical Sciences, Nazarbayev University School of Medicine, Astana, Kazakhstan; Nigerian Institute of Medical Research, NIGERIA

## Abstract

**Background:**

Antiretroviral therapy (ART) effectiveness is compromised by the emergence of HIV drug resistance mutations (DRM) and can lead to the failure of ART. Apart from intrinsic viral factors, non-compliance with drugs and/or the use of sub-optimum therapy can lead to the emergence of DRMs. In Pakistan HIV currently exists as a concentrated epidemic, however, ART coverage is very low, and drug adherence is poor. ART is selected assuming without baseline genotyping. Pakistan has recently seen a rise in treatment failures, but the country’s actual burden of DRM is still unknown. In this study, we perform the genetic and drug resistance analysis of the pol gene from Pakistani HIV-positive ART-naïve and ART-experienced individuals.

**Methods:**

In this study, HIV-1 pol was sequenced from 146 HIV-1 positive individuals, divided into ART-naïve (n = 37) and ART-experienced (n = 109). The sequences were also used to determine HIV-1 subtypes, the prevalence of DRM, and pol genetic variability.

**Results:**

DRM analysis identified numerous DRMs against reverse transcriptase inhibitors in both ART-naïve and ART-experienced groups, including a few that are classified as rare. Additionally, the ART-experienced group showed mutations associated with resistance to protease inhibitors. Genetic analysis showed negative selection pressure in both groups, but a higher rate of evolution in the ART-naïve group.

**Conclusion:**

High prevalence of DRMs, especially against previous first-line treatment in ART- naïve and the accumulation of DRMs in ART-experienced groups is concerning and warrants that a more extensive DRM survey be carried out to inform first-line and second-line ART regimen recommendations.

## Introduction

Antiretroviral therapy (ART) [1] has successfully increased the survival and quality of life of people living with HIV (PLHIV) globally. However, the effectiveness of ART is greatly compromised by the emergence of drug resistance mutations (DRM) in the HIV genome [2, 3], leading to the failure of ART therapy [4]. The major viral factor contributing to HIV drug resistance is its error-prone reverse transcriptase activity that facilitates the occurrence of a large number of mutations [5]. Other viral factors contributing to the emergence of DRM include recombination between viral strains, subtype-specific polymorphisms, and the emergence of minor variants [6]. Additionally, non-viral factors such as non-adherence to therapy, use of sub-optimum regimens, and use of ineffective drugs (due to pre-existing DRMs in treatment naïve patients) also contribute to the emergence of DRM [6]. The latter warrants that baseline and periodic genotyping of patients be carried out to monitor the emergence of drug resistance and choose the most effective drug combination [7].

According to the World Health Organization (WHO), transmitted HIV drug resistance mutations are those that are observed in patients who are unexposed to ART, i.e. in ART-naïve patients [6]. Certain transmitted drug resistance mutations (TDRM) revert to wild type in absence of drug selection pressure, for example, T215Y/F. The occurrence of TDRMs in ART-naïve patients is a major concern as it can limit treatment options and efficacy and can also lead to virological failure [8, 9]. The prevalence of acquired and transmitted (primary) resistance varies geographically [10]. According to a recent report by WHO, drug resistance has greatly increased against efavirenz (EFV) and nevirapine (NVP), which were traditionally part of ART in 12 countries in Africa, Asia, and the Americas [11]. According to a WHO report (2017), the most common drug resistance mutations against reverse transcriptase inhibitors in high-prevalence countries, such as Cameroon, Namibia, Uganda, Zimbabwe, Argentina, Brazil, Colombia, Guatemala, Mexico, Nicaragua, and Myanmar were M41L, M184V/I, T215any, K103N/S, Y181 and G190any, among ART-naïve and M184V/I, K65R, K103N/S, Y181any and G190any among ART-experienced [6]. The protease inhibitor resistance mutations were extremely rare, with a frequency of 1.8% in ART-experienced and 0.2% in ART-naïve [6]. Conversely, in Kenya, 22% (14/22) of minor protease drug resistance mutation (L33I) was observed in naïve individuals [10]. Moreover, in Africa, Mexico, Central, and South America, and South-East Asia, the prevalence of DRMs associated with resistance to non-nucleoside reverse transcriptase inhibitor were found to be <10% only in ART-naïve individuals, while, it was considerably higher (21.6%) among ART-experienced [6].

In Pakistan, HIV exists as a concentrated epidemic, with high prevalence in certain high-risk groups, such as people who inject drugs (38.4%), followed by male sex workers (5.6%), female sex workers (2.2%), transgender sex workers (7.5%), men who have sex with men (5.4%) and transgender people (7.1%) [12, 13]. The number of PLHIV has increased drastically since the beginning of the epidemic to 0.21 million in 2022, involving numerous HIV outbreaks such as the unprecedented 2019 HIV-1 outbreak in children that occurred in the Larkana district of Sindh province [14–16].

Currently, in Pakistan, the first-line regimen is a backbone of tenofovir (TDF) and lamivudine (3TC) with dolutegravir (DTG) though both zidovudine (AZT) and abacavir (ABC) may be used in special circumstances [17]. ART is prescribed in Pakistan without performing baseline or periodic sequencing, increasing the possibility that cases may be on ineffective therapy. The data on HIV DRMs in Pakistan is very limited; only two studies had reported a high prevalence of drug resistance mutations in ART-naïve and ART-experienced Pakistani PLHIV [18, 19]. No data exist on DRMs in Pakistani PLHIV with virological failure.

The current study presents genetic and drug resistance analysis of samples collected from ART-naïve and ART-experienced (including virological failure) Pakistani PLHIV before the rollout of DTG-based regimen. It is important to mention that the analysis of DRMs associated with reverse transcriptase (RTIs) and protease inhibitors (PIs) continues to hold significance in PLHIV, for example detecting instances of drug resistance, in individuals that may not qualify for a switch or initiation on DTG due to reasons such as diabetes/hyperglycemia [20–22].

## Materials and methods

### Study design and PLHIV profile

This was a cross-sectional study, where approximately 159 blood samples were collected from Pakistani PLHIV based on a convenient sampling strategy, involving all PLHIV visiting the treatment centers during 2019–20 (study period). The samples were collected from Bridge Consultants Foundation, Karachi, Aga Khan University, Karachi, and Shaukat Khanum Memorial Hospital, Lahore. Written informed consent was obtained from all study participants before carrying out any study procedures. The study was approved by the Aga Khan University Ethical Review Committee (2019-1124-3195). In addition, a questionnaire was used to obtain demographics and relevant clinical information from the study participants’ medical records, which was used to further characterize the participants based on exposure, viral load, CD4 count and history of drug, etc.

Out of 159 samples, only 146 were used, while rest with short/poor sequence reads (obtained after sequencing) were removed from this study. The study participants were divided into two categories: ART-naïve (n = 37), described as those who had no history of ART at the time of sample collection, and ART-experienced (n = 109) who were on ART. The most recent viral loads of ART-experienced individuals were taken from their record, while viral loads for ART-naïve were performed as part of another project, and this record was also included in the study.

### DNA extraction and PCR amplification

Viral nucleic acid was extracted from all the PLHIV samples by using Qiagen’s QIAamp DNA blood mini kit according to the manufacturer’s instructions. The DNA was stored at -80 C till further use. The Thermo Scientific NanoDrop 2000/2000c spectrophotometer was used to determine the quality and quantity of the DNA. The samples with the concentration ≥20 ng/ul and ratio of 1.8 at 260/280 nm absorbance were selected for PCR.

Partial *Pol* (covering protease and reverse transcriptase region) gene was amplified from each blood sample using two sets of primer in two steps nested PCR strategy. For this purpose, two sets of outer primers were used, forward (POLOF CAGCATGYCAGGGAGTRGGRGGACC, nt; 1832–1856, HXB2, IBF1 5’-AAATGATGACAGCATGTCAGGGAGT-3’. nt 1823–1847, HXB2) and Reverse (IBR1 5’-AACTTCTGTATATCATTGACAGTCCA-3’. nt 3303–3328, HXB2) [23, 24]. Subsequently, 70ug DNA template was used in the first round and 2ul of its product was used as a template for a second round with primer set, forward (POLIF 5’-AGGCTAATTTTTTAGGGAARATYTGGCCTTCC-3’. nt 2078–2109, HXB2) and reverse (RTOUT3 5’-TATGTCATTGACAGTCCAGCT-3’. nt 3300–3320, HXB2). PCR Mastermix (ABM) Bestaq (2X) cat# G464 (9ul) and Hotstart (2X) cat# G906 (10ul) were used to prepare a 25ul reaction mixture with primer 0.8 pmol and 0.6 pmol for the first and second rounds, respectively. For conventional PCR, the Eppendorf Vapo.protect Mastercycler® Pro was used. Thermo cycle conditions were as follows: denaturation at 95°C for 5 min, followed by 40 cycles of denaturation at 95°C for 1 min, annealing at 50°C for IBF1/IBR1 and 55°C for POLOF/IBR1 sets (round 1), 60°C (round 2) for 20 seconds, extension at 72°C for 1 min with a final extension of at 72°C for 7 min. The amplified products were then subsequently used for sequencing by the Sanger sequencing platform from Macrogen, Korea. Among 159 blood samples, only 146 were sequenced successfully, rest of them which had short/poor reads were removed from the study. The sequences used in this study were deposited in GenBank and were assigned the accession numbers MT223535-MT223672, MT240849, MT240850, MT240851, MT240852, MH021597, MK927053, MN549354, and MN385421.

It is important to mention that only protease and reverse transcriptase regions were evaluated in these PLHIV as none of the participants were on integrase inhibitors at the time of the study.

### Sequence alignment and HIV-1 genotyping

The obtained sequences were aligned using MEGA 6 software version 7.0. The alignment was cleaned to remove non-nucleotide characters. For HIV-1 subtyping, the pol sequences were processed through the REGA HIV online subtyping tool [25].

### Analysis of drug resistance mutations and site-specific genomic variability

HIV-1 pol gene (protease and reverse transcriptase) was analyzed using the Stanford University HIV Drug Resistance Database HIVdb algorithm [26, 27] and confirmed using the 2019 Update of the Drug Resistance Mutations in HIV-1 by the International AIDS Society–USA (IAS–USA) [28]. The DRMs were classified as high, intermediate, low, and potential low-level resistance, based on the information described in the Stanford University HIV Drug Resistance Database HIVdb algorithm and IAS-USA report [26, 29]. The prevalence of transmitted drug resistance was analyzed by using the surveillance drug resistance mutations (SDRM) list recommended by the WHO in 2009, among the ART-naïve PLHIV in our study cohort [30].

### Genomic variability, selection pressure, and rate variation on DRM sites

Genomic variability on DRM sites in pol sequences was analyzed in ART-naïve and ART-experienced groups using the Shannon entropy analysis tool available at the Los Alamos National Laboratory HIV Sequence Database [31]. We estimated the probability of DRM sites under negative and positive selection pressure in ART-naïve and ART-experienced groups using the FUBAR method [32]. Finally, we used the TreeRate tool using the Los Alamos National Laboratory HIV Sequence Database [33, 34], to calculate the estimated evolutionary rate in ART-naïve and ART-experience groups, using the generalized midpoint optimization method [33, 34]. The statistical significance of the difference between the mean evolutionary rate of the two groups was calculated using an unpaired T-test using GraphPad Prism tool, where p<0.05 was considered significant.

## Results

### PLHIV profile

The demographic information collected from participants revealed that individuals belonged to different ethnic groups present in region of sample collection, including Punjabi (n = 42), Pathan (n = 24), Baloch (n = 20), and Muhajir (n = 15), along with other minorities (Table 1). Among these 146 study subjects, 125 were males and 21 were females, aged between 13 and 76 years (mean 36) (Table 1). Eighty-nine participants were married, while 40 were unmarried and one had divorced (Table 1). In the ART-naïve group, three participants reported that their partners were HIV positive, while 12 reported their partners to be HIV negative. Similarly, in the ART-experienced group, 28 participants reported that their partners were HIV positive, while 35 reported their partners to be HIV negative; the rest of the participants were not aware of the HIV status of their partners (Table 1). Based on high-risk behavior, 65 participants were identified as PWID, followed by 11 and 10 participants with a history of sexual contact with CWSW (cisgender women sex workers) and MSM (men who have sex with men), respectively (Table 1).

**Table 1:** Demographic and clinical data of study participants. The table shows data pertaining to individuals’ ethnicities, gender, high-risk behaviors, etc., as well as their clinical parameters.

Parameters	Number of Participants n	
**Gender**		
Male	125	
Female	21	
**Age**		
mean age in years (age range)	36	
**Marital status**		
Married	89	
Single	40	
Divorced	1	
No record	16	
**Race/Ethnicity**		
Punjabi	42	
Pathan	24	
Baloch	20	
Mahajir	15	
Sindhi	10	
Bangali	6	
Memon	4	
Minorities	7	
No record	18	
**High-Risk Behaviors**		
PWID	65	
MSM	10	
CWSW	11	
Spouse	5	
Heterosexual	2	
PWID&CWSW	1	
Blood transfusion/Injections/injury	9	
Undeclare	12	
No record	31	
**ART history**		
Naïve	37	
Experienced	109	
**Partner’s HIV status**		
Concordant HIV status in ART-naïve	3	
Discordant HIV status in ART-naïve	12	
Concordant HIV status in ART-experienced	28	
Discordant HIV status in ART-experienced	35	
Don’t know	14	
No record	14	
**Viral load (copies/mL)**	**ART-naive**	**ART-experienced**
	n (%)	n (%)
undetectable	0	16
<500	7	1
500–999	0	5
1000–100000	13	45
>100000	0	16
no record	17	26 (
**CD4 count (cells/mm** ^**3**^ **)**	**ART-naive**	**ART-experienced**
	n (%)	n (%)
<100	0	7 (0.06)
>100-<400	8	32
>400-<500	10	30
>500	18	23
No record	1	17

**Fig 1:**
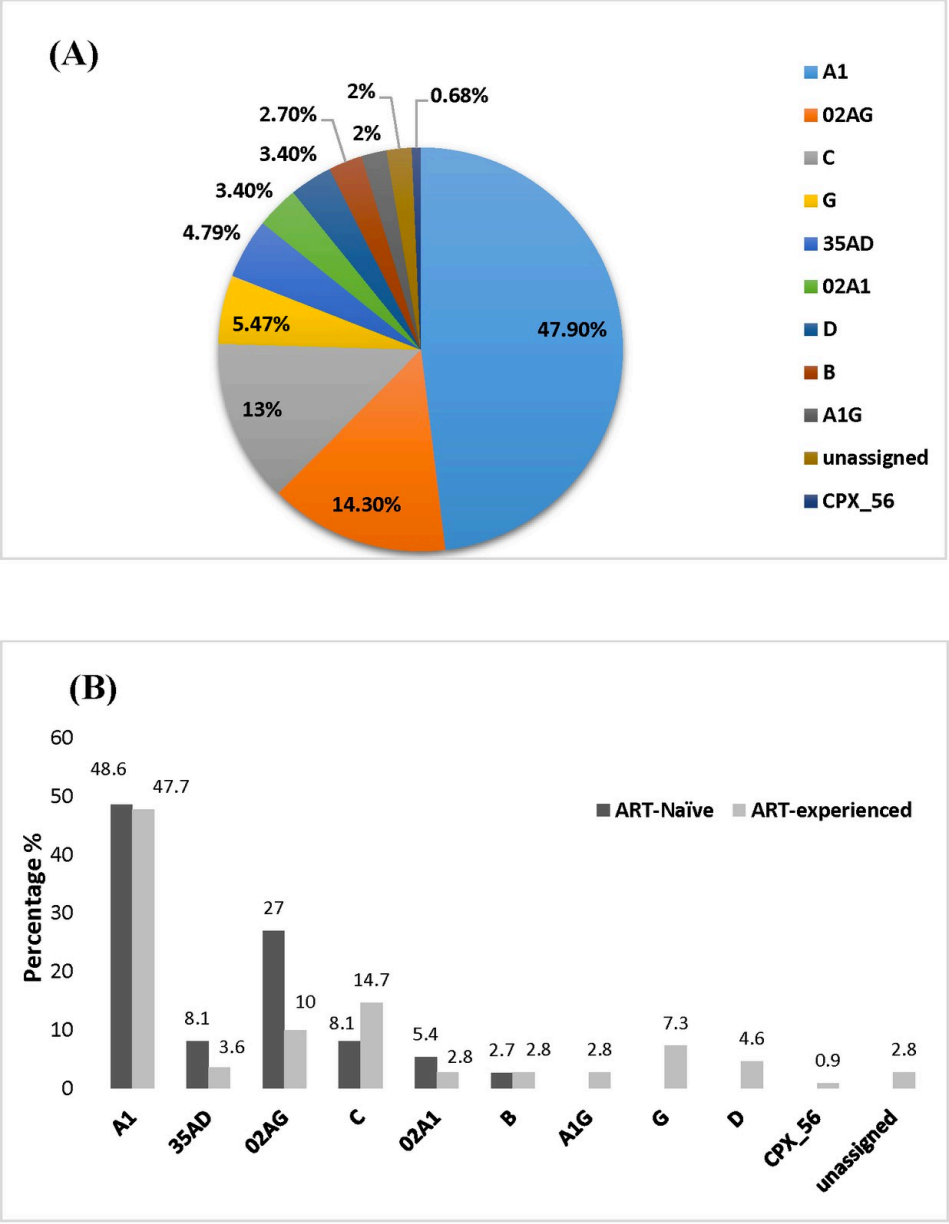
HIV subtype distribution. **A)** Frequency of HIV-1 subtype in all samples, **B)** HIV-1 subtype distribution in ART-naïve and ART-experienced group. Each bar represented the subtype frequency in each group.

We also divided patients based on CD4 counts and viral loads (Table 1). Based on CD4 counts, 7, 40, 40, and 41 individuals had CD4 counts <100, >100 but <400, >400 but <500, and >500 cells/mm^3^, respectively, while 23 patients had no record of their CD4 counts (Fig 1). Similarly, 16 participants had undetectable viral loads (<200 copies/ml) while, the majority of the participants had high (1000 RNA copies/mL) to very high (>10,000 to >100,000 RNA copies/mL) viral loads; 43 participants had no records of their viral load (Table 1).

### HIV-1 subtyping

HIV-1 subtype analysis revealed the presence of diverse subtypes in our cohort. The predominant subtypes observed in our cohort were subtype A1 (47.9%), followed by subtype CRF_02AG (14.3%), C (13%), G (5.47%), and CRF_35AD (4.79%) (Fig 1A).

Comparative subtype analysis between the two groups (ART-naïve and ART-experienced) revealed subtype A1 to be the predominant subtype in both groups (Fig 1B). In addition, CRF_02AG, CRF_35AD, and 02A1 were predominant in the ART- naïve group, while, subtypes C, G, D, and A1G were predominant in the ART-experienced group (Fig 1B). Interestingly, unassigned subtypes, along with CRF_56cpx, were only observed in ART-experienced PLHIV with virologic failure (Fig 1B).

### Analysis of drug resistance mutations

Analysis of DRMs in the ART-naïve group showed a high prevalence of the following DRMs: E138A (38.8%) and K103N (10.8%), associated with resistance against NNRTIs; and K219E (15.6%), M184L (11.4%) and K70R (5.4%) associated with resistance against NRTI (Table 2). Similarly, a major mutation I84V (2.7%), and a minor mutation N83D (2.7%) conferring resistance against PI, were observed only in one patient each (Table 2).

**Table 2:** Classification of the drug resistance mutations. The table describes the frequency and classification of all DRMs in terms of the nature of the mutation, and their association with drug resistance as described in the Stanford University HIV Drug Resistance Database HIVdb algorithm (1) and the International Aids Society (IAS) 2019 report (38). In the mutation classification column, * indicates mutations that are classified as major and minor DRMs or others (no role identified) by the Stanford drug resistance database, while ˣ indicates mutations that are classified as major or minor DRMs by the International AIDS Society. In the drug associated with resistance column, * shows the association of a mutation with resistance to a particular drug, as indicated in the Stanford drug resistance database; while ˣ shows the association of a mutation with a particular drug, as indicated by the International AIDS Society (IAS) 2019 report, while bold drug names indicates the major resistance by IAS 2019. The abbreviations of the antiretroviral drugs are as follows: PI; atazanavir/ritonavir (ATV/r), darunavir/ ritonavir (DRV/r), fosamprenavir/ ritonavir (FPV/r), indinavir/ ritonavir (IDV/r), lopinavir/ ritonavir (LPV/r), nelfinavir (NFV), tipranavir/ ritonavir (TPV/r). NRTI; stavudine (d4T), didanosine (DDI), zidovudine (AZT), emtricitabine (FTC), lamivudine (3TC), abacavir (ABC), tenofovir (TDF). NNRTI; efavirenz (EFV), nevirapine (NVP), etravirine (ETR), rilpivirine (RPV), doravirine (DOR). The abbreviations used to classify the mutations are as follows: P = polymorphic, NP = non polymorphic, M = Major, m = Minor, U = unusual, HU = highly unusual, N = none, O = other, ER = extremely rare, TAM = thymidine analogue mutation, H = hypermutation, R = rare, CP = common polymorphic.

Drugs		Mutations	ART-naïve %	ART-experienced %	Mutation Classification	Drugs associated with resistance
**Protease inhibitor**	**Major**	**M46I**	0.0	1.8	*ˣM/NP or ˣm	ˣ[*(atv/r, lpv/r), fpv/r, nfv, **idv/r**]
		**I47V**	0.0	0.9	*ˣM/NP or ˣm	*(atv/r, lpv/r, drv/r),ˣ**(drv/r**, fpv/r, **lpv/r, tpv/r)**
		**I54V**	0.0	1.8	*M/NP or ˣm	ˣ[fpv/r, idv/r, sqv/r, tpv/r, *(atv/r, lpv/r)]
		**V82M**	0.0	0.9	*M	*atv/r, lpv/r
		**V82A**	0.0	0.9	*ˣM/NP or ˣm	ˣ [fpv/r, **idv/r,** nfv, sqv/r, *(atv/r, **lpv/r)**]
		**V82S**	0.0	0.9	*ˣM/NP or ˣm	ˣ(fpv/r, **lpv/r**, nfv, sqv/r)*(atv/r, lpv/r)
		**I84V**	2.7	0.9	*ˣM/NP or ˣm	**ˣ[fpv/r, idv/r, tpv/r**, nfv, sqv/r, *(**atv/r, drv/r,** lpv/r)]
	**Minor**	**T74P**	0.0	0.9	*ˣm/NP or ˣM	ˣ(drv/r, tpv/r), *atv/r
		**N83D**	2.7	0.0	*m/NP or ˣM	*(atv/r), **ˣ(tpv/r)**
		**K20T**	0.0	0.9	*ˣm/NP	ˣatv/r
		**V32L**	0.0	0.0	*m/HU	None
		**V32G**	0.0	0.0	*m/HU	None
		**V32E**	0.0	0.9	*m/HU	None
		**L24I**	0.0	0.9	*ˣm/NP	ˣ[idv/r, sqv/r, *(atv/r, lpv/r)]
		**G73R**	2.7	0.0	*m/U	None
		**I50F**	0.0	0.9	*m/HU	None
	**Other**	**L10V**	2.7	4.6	*O/P or ˣm	ˣatv/r, fpv/r, idv/r, lpv/r, sqv/r, tpv/r
		**L10I**	8.1	9.2	*O/P or ˣm	ˣatv/r, fpv/r, idv/r, lpv/r, nfv, sqv/r
		**K20R**	10.8	13.7	*O/HP or ˣm	ˣatv/r, idv/r, lpv/r
		**K20I**	32.4	27.5	*O or ˣm	ˣatv/r
		**D30H**	2.7	0.0	*O/HU	None
		**V82I**	13.5	11.9	*O/P or ˣm	ˣatv/r
		**F53Y**	0.0	0.9	*O/R, NP or ˣm	ˣatv/r
		**L90V**	0.0	0.9	*O/HU	None
		**L24S**	0.0	0.9	*O/HU	None
		**T74S**	0.0	1.8	*O/ P	None
		**V11I**	2.7	0.0	*O/NP or ˣmr	ˣdrv/r
		**A71V**	0.0	0.9	*O/P or ˣm	ˣatv/r, idv/r, lpv/r, nfv, sqv/r
**Reverse Transcriptase Inhibitors**	**NRTI’s**	**K70R**	5.4	4.6	ˣM	* [ddi, abc, tdf, ˣ(**azt, d4t**)]
		**K70G**	2.7	0.0	*N	*3tc, ftc, abc, tdf
		**K70E**	0.0	2.7	ˣM	*[3tc, ftc, abc, ˣ(**tdf**)]
		**K70Q**	0.0	0.0	*N	*3tc, ftc, abc, tdf
		**Y115F**	0.0	0.0	ˣM	*[tdf, ˣ(**abc**)]
		**K219E**	15.6	4.7	*N or ˣM	**ˣ[d4t,** *azt]
		**K219Q**	3.1	2.8	*N or ˣM	**ˣ[d4t,** *azt]
		**K219N**	0.0	1.8	*N	*azt
		**M184V**	0.0	12.8	ˣM	*ˣ**abc, ftc, 3tc**
		**M184L**	11.4	4.6	*HU	None
		**M184I**	0.0	1.8	*N or ˣM	*[abc**,ˣ**(**ftc, 3tc**)
		**M184W**	2.8	0.0	*HU	None
		**M184P**	2.8	0.9	*HU	None
		**M184T**	2.8	0.0	*HU	None
		**M184E**	0.0	0.9	*HU	None
		**L74V**	0.0	0.9	*N or ˣM	*ˣ**abc, ddi**
		**L74I**	0.0	0.9	*N	*abc, ddi
		**F77L**	0.0	0.9	*N or ˣM	ˣ[d4t, ddi, ftc, 3tc, abc, *(azt)]
		**M41L**	0.0	2.8	*TAM or ˣM	ˣ[d4t, *azt]
		**D67K**	2.7	0.0	*HU	None
		**D67N**	0.0	4.6	*NP or ˣM	*ˣ**d4t, azt**
		**D67G**	0.0	0.9	*NP	*azt
		**T69D**	0.0	0.9	*NP	*ddi, d4t
		**V75M**	0.0	1.8	*N or ˣM	ˣ[**ftc, 3tc, abc,** *(**azt, d4t, ddi**)]
		**K65E**	2.7	0.0	*ER or ˣM	ˣddi, ftc, 3tc, d4t, *(**tdf, abc**)
		**K65R**	0.0	2.8	*N or ˣM	ˣ[**ddi, d4t,** *(**tdf, ftc, 3tc, abc**)]
		**T215Y**	0.0	0.9	*TAM or ˣM	*[abc, tdf, ddi, **ˣ**(**d4t, azt**)]
		**T215A**	3.1	0.9	*N	*azt
		**T215V**	0.0	0.9	*N	*azt
		**T215F**	0.0	0.9	*TAM or ˣM	*[abc, tdf, ddi, **ˣ**(**d4t, azt**)]
		**T215S**	0.0	0.9	*N	*azt
		**T215I**	0.0	0.9	*N	*azt
		**L210W**	0.0	0.9	*TAM or ˣM	ˣ[d4t, *azt]
	**NNRTI’s**	**E138A**	38.8	35.0	*P or ˣM/ m	*ˣetr, **rpv**
		**Y181F**	0.0	1.8	*NP(R)	*nvp, rpv
		**Y181C**	0.0	2.8	*NP or ˣM	*[dor,ˣ(**efv, etr, nvp, rpv**)]
		**Y181H**	2.8	0.0	*HU	None
		**K103N**	10.8	19.2	*NP or ˣM	*ˣ**efv, nvp**
		**K103T**	0.0	0.9	*NP/ER	*efv, nvp
		**K103E**	2.7	0.0	*R	None
		**V179T**	0.0	5.5	*NP or ˣm	*(rpv,ˣetr)
		**V179D**	0.0	3.6	*P or ˣm	*[efv, nvp, rpv, ˣ(etr)]
		**V179F**	0.0	0.9	*NP or ˣm	*[efv, nvp, rpv, ˣ(etr)]
		**K101E**	0.0	2.8	*NP or ˣM/m	*[nvp, efv, dor, ˣ(etr, **rpv**)]
		**K101P**	0.0	1.8	*NP or ˣM	*[dor, ˣ(**efv, etr, nvp, rpv**)]
		**G190A**	2.9	6.4	*NP or ˣM/ m	*[rpv, ˣ(**efv,** etr, **nvp**)]
		**G190E**	0.0	0.9	*NP	*dor, efv, nvp, rpv, etr
		**G190P**	0.0	0.9	*HU	none
		**G190Q**	0.0	0.9	*NP	*dor, efv, nvp, etr, rpv
		**G190S**	0.0	0.9	*NP or ˣM/m	*[rpv, nvp, dor, **ˣ**(**efv,** etr)]
		**F227L**	0.0	2.8	*NP	*nvp, dor, efv
		**F227I**	0.0	0.9	*ER	*nvp, dor, efv
		**F227C**	3.2	0.0	*NP or ˣM	*dor, efv, etr, nvp, ˣ**rpv**
		**P225H**	0.0	0.9	*NP or ˣM	*nvp, dor, ˣ**efv**
		**V106A**	2.7	0.0	*NP or ˣM	*[efv, dor, ˣ(**nvp**)]
		**V106I**	0.0	1.8	*N or ˣm	*[(nvp, rpv, dor, ˣ(etr)]
		**V106M**	0.0	1.8	*NP or ˣM	*ˣ**nvp, dor, efv**
		**V108I**	0.0	1.8	*NP or ˣM	*[dor, ˣ(**efv, nvp**)]
		**M230L**	0.0	1.8	*NP or ˣM/m	*[dor, ˣ(**efv,** etr**, nvp, rpv**)]
		**M230I**	3.3	0.0	*ER or ˣM	*[nvp, dor, efv, etr, ˣ(**rpv**)]
		**Y188N**	0.0	0.9	*HU	None
		**Y188F**	0.0	0.9	*NP (R)	*nvp, efv, dor, rpv
		**Y188L**	0.0	2.8	*NP or ˣM	*[etr, dor, ˣ(**efv, nvp, rpv**)]
		**Y188D**	0.0	1.8	*HU	none
		**Y188S**	0.0	0.9	*HU	none
		**Y188P**	2.9	0.0	*HU	none
		**Y188H**	2.9	0.0	*NP or ˣM	*[efv, dor, **ˣ**(**nvp**)]
		**L100I**	0.0	0.9	*NP or ˣM	*[dor, ˣ(**efv, etr, nvp, rpv**)]
		**L100F**	0.0	0.9	*HU	none
		**H221Y**	0.0	1.8	*NP or ˣM	*[dor, efv, etr, nvp,ˣ(**rpv**)]
		**A98G**	0.0	0.9	*NP or ˣm	*[dor, efv, rpv, nvp, ˣ(etr)]
		**K238T**	0.0	0.9	*NP	*efv, nvp
	**Other**	**F116L**	5.4	3.6	*O/ HU	None
		**V118I**	0.0	4.6	*O/P	None
		**V179A**	0.0	0.9	*O/U	None
		**V179I**	54.2	52.2	*O/P	None
		**V179Y**	0.0	0.9	*O/U	None
		**K238E**	17.2	7.3	*O/ HU	None
		**K238R**	3.4	2.8	*O/ CP	None
		**K238S**	3.4	0.0	*O/ HU	None
		**K238I**	0.0	1.8	*O/ HU	None
		**K238Q**	0.0	2.8	*O/ HU	None
		**K103R**	8.1	1.8	*O/P	None
		**S68G**	0.0	1.8	*O/P	None
		**T69N**	2.7	4.6	*O/NP	None
		**T69A**	0.0	0.9	*O/P	None
		**T69S**	0.0	1.8	*O/P	None
		**V90I**	5.4	4.6	*O/P or ˣm	ˣetr
		**K101N**	2.7	0.0	*O/NP	None
		**M230R**	0.0	0.9	*O/ HU	None
		**M230K**	6.6	2.8	*O/ HU	None
		**M230E**	0.0	0.9	*O/ HU	None
		**F227H**	0.0	0.9	*O/ HU	None
		**F227Y**	3.2	0.9	*O/ HU	None
		**K219I**	0.0	0.9	*O/ U	None
		**K219T**	0.0	1.8	*O/ U	None
		**K219L**	6.2	0.0	*O/ U	None
		**K219Y**	6.2	0.9	*O/ U	None
		**K219D**	0.0	0.9	*O/ U	None
		**K219S**	0.0	0.9	*O/ U	None
		**V106L**	8.1	2.8	*O/ HU	None
		**V108E**	0.0	0.9	*O/ HU	None
		**Y115H**	2.7	0.9	*O/ HU	None
		**P225L**	3.2	1.8	*O/ HU	None
		**P225T**	3.2	0.0	*O/ HU	None
		**P225A**	0.0	0.9	*O/ HU	None
		**P236S**	0.0	0.9	*O/ HU	None
		**L234P**	0.0	0.9	*O/ HU	None
		**E183P**	0.0	0.9	*O/ U	None
		**G190R**	0.0	1.8	*H	None
		**Q151R**	0.0	0.9	*O/ HU	None
		**V179G**	0.0	0.9	*O/ HU	None

DRM analysis of the ART-experienced group showed a high prevalence of the following DRMs: E138A (35%), K103N (19.2%), G190A (6.4%), and V179T (5.5%), conferring resistance against NNRTIs; and M184V (12.8%), K219E (4.7%), M184L (4.6%), K70R (4.6%) and D67N (4.6%) conferring resistance against NRTI (Table 2). Additionally, the coexistence of DRM M46I (1.8%) and I54V (1.8%) was observed in two patients; while I47V and V82M/A/S and I84V were seen in one patient each, conferring resistance against PI (Table 2).

Interestingly, certain mutations classified as rare/unusual/other mutations by the HIV Stanford drug resistance database (1) were found in high frequency in both the study groups (Table 2). Highly prevalent mutations observed in the ART-naïve group were: V179I (54.2%), K238E (17.2%), K103R (8.1%), V106L (8.1%), M230K (6.6%), K219L/Y (6.2%) and F116L (5.4%) in reverse transcriptase region, while K20I (32.4%), K20R (10.8%), V82I (13.5%), and L10I (8.1%) in the protease region (Table 2). Similarly, the mutations predominant in the ART-experienced group were V179I (52.2%), K238E (7.3%), V90I (4.6%), T69N (4.6%), and V118I (4.6%), in reverse transcriptase region, and K20I (27.5%), K20R (13.7%), V82I (11.9%), L10I (9.2%) and L10V (4.6%) in the protease region in high prevalence (Table 2).

**Fig 2:**
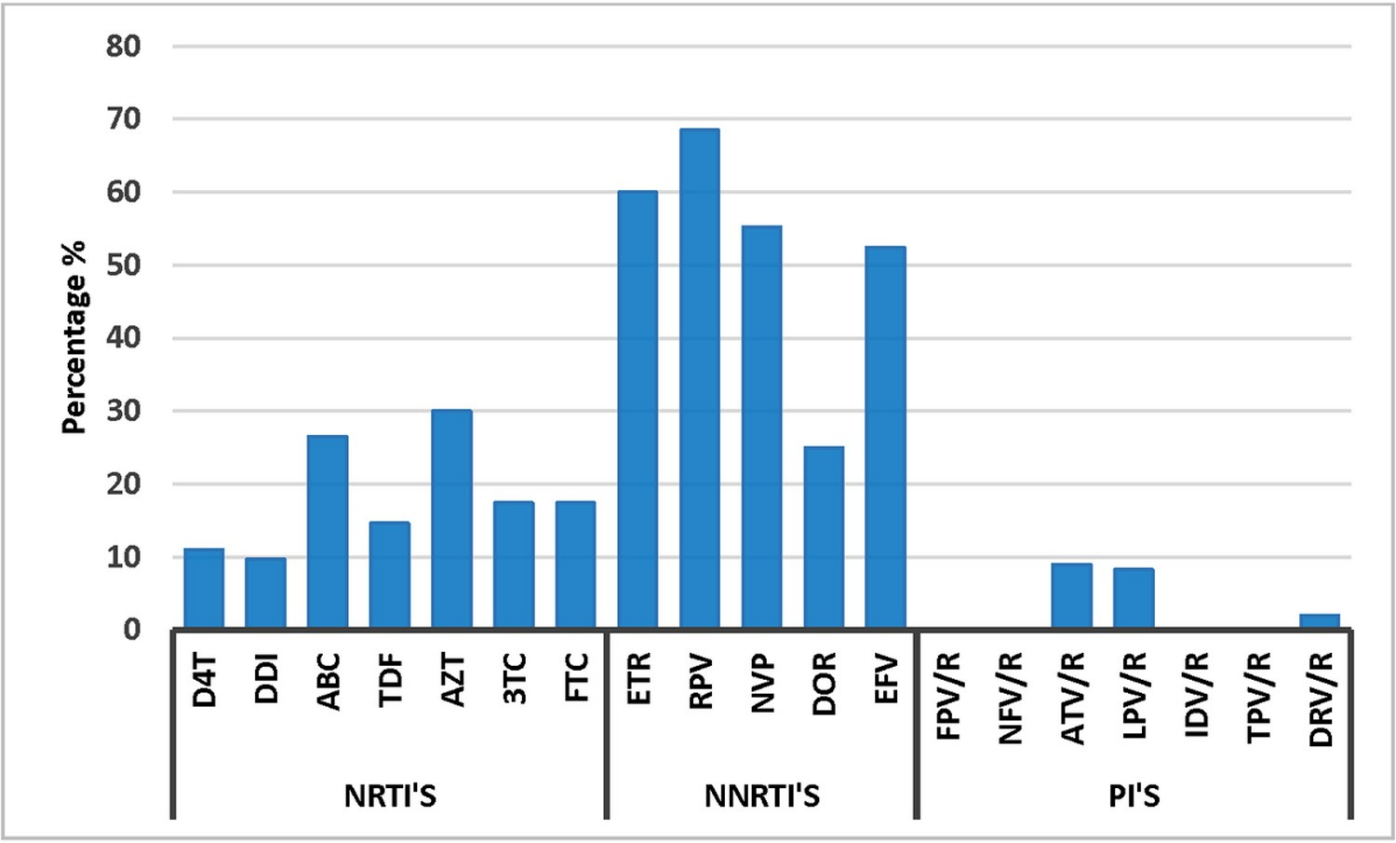
Prevalence of drug resistance against NRTI, NNRTI and PI. **Abbreviations:** PI; atazanavir/ritonavir (ATV/r), darunavir/ ritonavir (DRV/r), fosamprenavir/ ritonavir (FPV/r), indinavir/ ritonavir (IDV/r), lopinavir/ ritonavir (LPV/r), nelfinavir (NFV), tipranavir/ ritonavir (TPV/r). NRTI; stavudin (d4T), didanosin (DDI), zidovudin (AZT), emtricitabine (FTC), lamivudine(3TC), abacavir (ABC), tenofovir (TDF). NNRTI; efavirenz (EFV), nevirapine (NVP), etravirine (ETR), rilpivirine (RPV), doravirine (DOR).

Analysis of DRM on ART efficacy showed that the drugs found to be most affected by DRMs were rilpivirine (68.5%), etravirine (60%), nevirapine (55.2), and efavirenz (52.4%) in NNRTI class; zidovudine (30%) and abacavir (26.5%) in NRTI class; and atazanavir/ritonavir (9%) and lopinavir/ritonavir (8.3%) in PI class (Fig 2).

### Genomic variability, selection pressure, and rate variation on DRM sites

Entropy (variability) analysis of the DRM sites in the ART-naïve group revealed DRM sites 20, 103, 138, 179, 184, 210, 219, 221, 225, 227, 230, and 238 had high entropy score which was greater than the mean entropy value (0.28) in ART-naïve group. Similarly, in the ART-experienced group, DRM codon sites 20, 103, 138, 179, 184, 190, 219, 221, 230, and 238 exhibited high entropy score, which was greater than the mean entropy score (0.4) observed in the ART-experienced group (Table 3).

**Table 3:** Drug resistance mutations present in study participants. The table shows SDRMs in column 2, marked with an asterisk (*), and frequency of DRMs (columns 3 and 4), entropy score (column 5), and selection pressure (column 6) for each drug resistance mutations sites in both ART-naïve and ART-experienced groups. In column 5, the grey shaded column indicates high entropy sites, the entropy score which was greater than the mean value characterized as high, and the entropy score less than the mean value characterized as low entropy in ART-naïve (mean = 0.28) and ART-experienced (mean = 0.4) group, while ‘0’ represents no entropy. In column 6,—values and + values indicate the probability of negative and positive selection pressure on DRM sites, respectively, whereas, the bold and underlined values show high selection pressure between ART-naïve and ART-experienced groups.

Drugs		Mutations	ARTnaïve (%)	ART-experienced (%)	Entropy		Selection pressure	
					ART-naïve	ART-experienced	ART-naïve	ART-experienced
**Protease inhibitor**	**Major**	**M46I**	0.0	1.8	0.00	0.18	-0.93	-0.95
		**I47V**	0.0	0.9	0.00	0.14	-0.86	-0.80
		**I54V**	0.0	1.8	0.00	0.18	-0.95	-0.99
		**V82M**	0.0	0.9	0.44	0.60	0.63	0.62
		**V82A**	0.0	0.9				
		**V82S**	0.0	0.9				
		*I84V	2.7	0.9	0.12	0.14	-0.65	-0.80
	**Minor**	**T74P**	0.0	0.9	0.00	0.24	-0.79	-0.87
		**N83D**	2.7	0.0	0.25	0.29	-0.84	-0.79
		**K20T**	0.0	0.9	1.04	1.11	-0.64	**-0.85**
		**V32L**	0.0	0.0	0.00	0.28	-1.00	-0.98
		**V32G**	0.0	0.0				
		**V32E**	0.0	0.9				
		**L24I**	0.0	0.9	0.00	0.23	-0.97	-0.88
		**G73R**	2.7	0.0	0.12	0.09	-0.79	**-1.00**
		**I50F**	0.0	0.9	0.00	0.14	-0.78	-0.58
	**Other**	**L10V**	2.7	4.6	0.52	0.66	-0.97	-0.86
		**L10I**	8.1	9.2				
		**K20R**	10.8	13.7	1.04	1.11	-0.64	**-0.85**
		**K20I**	32.4	27.5				
		**D30H**	2.7	0.0	0.12	0.14	-0.65	**-1.00**
		**V82I**	13.5	11.9	0.44	0.61	0.64	0.62
		**F53Y**	0.0	0.9	0.00	0.19	-1.00	-1.00
		**L90V**	0.0	0.9	0.12	0.10	0.67	-0.77
		**L24S**	0.0	0.9	0.00	0.23	-0.97	-0.88
		**T74S**	0.0	1.8	0.00	0.24	-0.79	**-0.88**
		**V11I**	2.7	0.0	0.12	0.00	-0.81	-1.00
		**A71V**	0.0	0.9	0.00	0.14	-0.98	-1.00
**Reverse Transcriptase Inhibitors**	**NRTI’s**	*K70R	5.4	4.6	0.33	0.52	-0.81	-0.97
		**K70G**	2.7	0.0				
		**K70E**	0.0	2.7				
		**K70Q**	0.0	0.0				
		**Y115F**	0.0	0.0	0.12	0.35	-0.60	**-0.98**
		*K219E	15.6	4.7	1.08	1.09	0.43	0.12
		**K219Q**	3.1	2.8				
		**K219N**	0.0	1.8				
		**M184V**	0.0	12.8	0.87	1.03	0.55	0.76
		**M184L**	11.4	4.6				
		**M184I**	0.0	1.8				
		**M184W**	2.8	0.0				
		**M184P**	2.8	0.9				
		**M184T**	2.8	0.0				
		**M184E**	0.0	0.9				
		**L74V**	0.0	0.9	0.00	0.23	-0.87	-0.93
		**L74I**	0.0	0.9				
		**F77L**	0.0	0.9	0.00	0.18	-1.00	-1.00
		**M41L**	0.0	2.8	0.00	0.32	-0.94	-0.84
		**D67K**	2.7	0.0	0.12	0.36	-1.00	-1.00
		**D67N**	0.0	4.6				
		**D67G**	0.0	0.9				
		**T69D**	0.0	0.9	0.12	0.50	-0.79	0.40
		**V75M**	0.0	1.8	0.00	0.31	**-0.94**	-0.79
		**K65E**	2.7	0.0	0.12	0.25	-0.93	-0.95
		**K65R**	0.0	2.8				
		**T215Y**	0.0	0.9	0.52	0.75	-0.88	-0.90
		**T215A**	3.1	0.9				
		**T215V**	0.0	0.9				
		**T215F**	0.0	0.9				
		**T215S**	0.0	0.9				
		**T215I**	0.0	0.9				
		**L210W**	0.0	0.9	0.64	0.62	-0.94	-0.99
	**NNRTI’s**	**E138A**	38.8	35.0	0.77	0.88	-0.77	**-1.00**
		**Y181F**	0.0	1.8	0.25	0.51	-0.82	-1.00
		**Y181C**	0.0	2.8				
		**Y181H**	2.8	0.0				
		*K103N	10.8	19.2	0.73	0.80	-0.38	-0.22
		**K103T**	0.0	0.9				
		**K103E**	2.7	0.0				
		**V179T**	0.0	5.5	0.79	1.42	**-0.86**	-0.04
		**V179D**	0.0	3.6				
		**V179F**	0.0	0.9				
		**K101E**	0.0	2.8	0.12	0.45	-0.82	-0.97
		**K101P**	0.0	1.8				
		*G190A	2.9	6.4	0.33	0.81	-0.53	**-0.89**
		**G190E**	0.0	0.9				
		**G190P**	0.0	0.9				
		**G190Q**	0.0	0.9				
		**G190S**	0.0	0.9				
		**F227L**	0.0	2.8	0.68	0.69	-0.79	**-0.97**
		**F227I**	0.0	0.9				
		**F227C**	3.2	0.0				
		**P225H**	0.0	0.9	0.68	0.61	-1.00	-1.00
		*V106A	2.7	0.0	0.40	0.46	-0.64	**-0.98**
		**V106I**	0.0	1.8				
		**V106M**	0.0	1.8				
		**V108I**	0.0	1.8	0.00	0.35	-0.99	-0.98
		**M230L**	0.0	1.8	0.83	0.90	-0.51	-0.70
		**M230I**	3.3	0.0				
		**Y188N**	0.0	0.9	0.46	0.65	-0.66	-0.60
		**Y188F**	0.0	0.9				
		**Y188L**	0.0	2.8				
		**Y188D**	0.0	1.8				
		**Y188S**	0.0	0.9				
		**Y188P**	2.9	0.0				
		*Y188H	2.9	0.0				
		**L100I**	0.0	0.9	0.00	0.23	-0.97	-0.95
		**L100F**	0.0	0.9				
		**H221Y**	0.0	1.8	0.99	0.94	0.66	-0.84
		**A98G**	0.0	0.9	0.00	0.36	-0.99	-1.00
		**K238T**	0.0	0.9	1.33	1.27	-0.46	-0.05
	**Other**	**F116L**	5.4	3.6	0.21	0.31	-0.99	-1.00
		**V118I**	0.0	4.6	0.40	0.57	-0.73	**-1.00**
		**V179A**	0.0	0.9	0.79	1.42	**-0.86**	-0.04
		**V179I**	54.2	52.2				
		**V179Y**	0.0	0.9				
		**K238E**	17.2	7.3	1.33	1.27	-0.46	-0.05
		**K238R**	3.4	2.8				
		**K238S**	3.4	0.0				
		**K238I**	0.0	1.8				
		**K238Q**	0.0	2.8				
		**K103R**	8.1	1.8	0.73	0.80	-0.38	-0.22
		**S68G**	0.0	1.8	0.12	0.32	-1.00	-1.00
		**T69N**	2.7	4.6	0.12	0.50	**-0.79**	0.40
		**T69A**	0.0	0.9				
		**T69S**	0.0	1.8				
		**V90I**	5.4	4.6	0.28	0.39	-0.93	-0.97
		**K101N**	2.7	0.0	0.12	0.45	-0.82	-0.97
		**M230R**	0.0	0.9	0.83	0.90	-0.51	-0.70
		**M230K**	6.6	2.8				
		**M230E**	0.0	0.9				
		**F227H**	0.0	0.9	0.68	0.69	-0.79	**-0.97**
		**F227Y**	3.2	0.9				
		**K219I**	0.0	0.9	1.08	1.10	0.43	0.12
		**K219T**	0.0	1.8				
		**K219L**	6.2	0.0				
		**K219Y**	6.2	0.9				
		**K219D**	0.0	0.9				
		**K219S**	0.0	0.9				
		**V106L**	8.1	2.8	0.40	0.46	-0.64	**-0.98**
		**V108E**	0.0	0.9	0.00	0.35	-0.99	-0.98
		**Y115H**	2.7	0.9	0.12	0.35	-0.60	**-0.98**
		**P225L**	3.2	1.8	0.68	0.61	-1.00	-1.00
		**P225T**	3.2	0.0				
		**P225A**	0.0	0.9				
		**P236S**	0.0	0.9	0.56	0.59	-1.00	-1.00
		**L234P**	0.0	0.9	0.44	0.55	-0.82	-0.92
		**E183P**	0.0	0.9	0.25	0.31	-0.75	**-1.00**
		**G190R**	0.0	1.8	0.33	0.81	-0.53	**-0.89**
		**Q151R**	0.0	0.9	0.00	0.29	-0.88	-1.00
		**V179G**	0.0	0.9	0.79	1.42	**-0.86**	-0.04

Similarly, selection pressure analysis of the DRM sites revealed sites 82, 184, and 219 to be under positive selection pressure, while all other DRM sites were negatively selected. Comparative analysis of DRM sites between ART-naïve and ART-experienced groups revealed DRM sites 75, 69, and 179 to be under high negative selection pressure in the ART-naïve group, while sites 20, 30, 73, 74, 106, 115, 118, 138, 183, 190, 227 to be under high negative selection pressure in the ART-experienced group. Additionally, DRM sites 90 and 221 were under positive and negative selection pressure in ART-naïve and ART-experienced group, respectively. Similarly, DRM site 69 was observed under negative selection in the ART-naïve group, whereas, neutral in the ART-experienced group. Out of the positively selected sites, DRM sites, 82 and 184 were under high positive selection pressure (Table 3).

**Fig 3:**
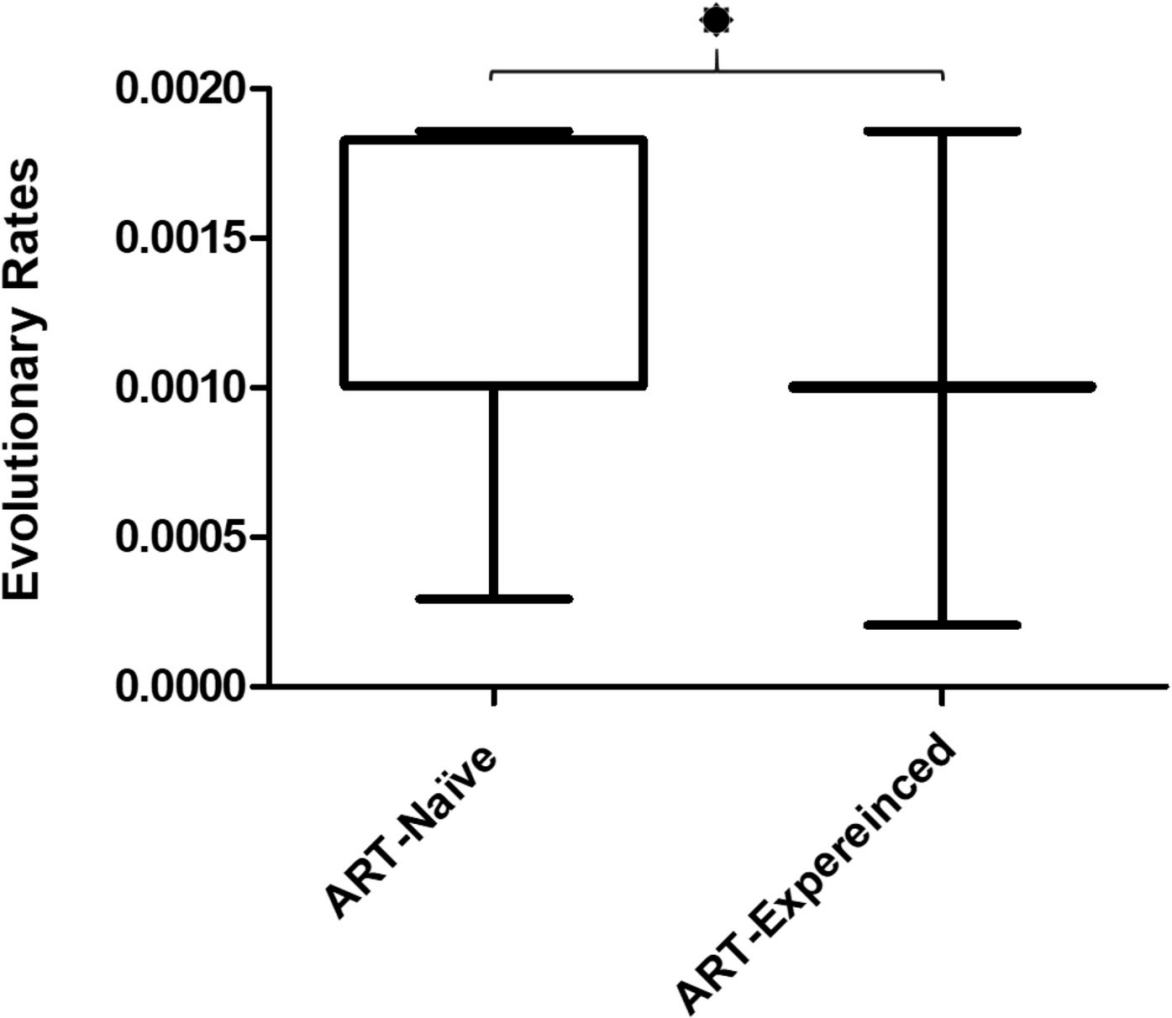
Evolutionary rate of the sequences from ART-naïve and ART-experienced groups. The graph shows the mean evolutionary rate and dispersion of rate in the ART-naïve and ART-experienced groups. The asterisk indicates statistical significance (p<0.05) in the mean evolutionary rate of the two groups.

Estimates of the evolutionary rates between the ART-naïve and ART-experienced groups suggested that the mean evolutionary rate of sequences in the ART-naïve group was significantly higher (0.001206 ± 0.00008259) than the ART-experienced group (0.001047 ± 0.00002795), and the difference in the mean evolutionary rate was statistically significant (p = 0.0445; Fig 3).

## Discussion

In this study, we performed the genetic and drug resistance mutation analysis of *pol* from a cohort of ART-naïve and ART-experience in Pakistani PLHIV.

The ethnic profile of our study participants suggested that most participants were Punjabis. This finding is consistent with previous reports which showed Punjabi and Sindhi to be the predominant ethnic groups among the PLHIV in Pakistan [35]. The participant profiles also suggested that the majority of PLHIV were males and PWIDs. This finding is also supported by previous studies showing that male PWIDs comprise the largest group in terms of HIV prevalence, followed by MSM and Hijra Sex workers (HSWs) [35]. We observed a large number of individuals with discordant HIV-1 status in both groups. This is not surprising as social stigmatization has forced many PLHIV in Pakistan to hide their HIV status from their partners [36]. In our cohort, the majority of our participants had viral loads greater than 1000 copies/ml in both ART-naïve (35%) and ART-experienced (41%) groups, respectively (Table 1) and can be categorized as possible virological failures if the observed viral load was >1000 copies/ml in two consecutive viral load test three months apart with adherence support following the first viral load measurement, as per WHO criteria [9]. As the ART adherence rate is poor [37], especially in the PWID which forms a large proportion of PLHIV these individuals may have drug-resistance viral strains, leading to therapy failure [37]. This is consistence with our observations where ART-experienced patients with high viral loads were found to be infected by drug-resistance recombinant strains of HIV [23, 38].

Analysis of subtype prevalence revealed a high prevalence of subtype A1 and CRF_02AG in our cohort (Fig 1A). This analysis is consistent with the previous studies where subtype A has consistently been shown to be the predominant subtype in Pakistan [39, 40], while CRF_02AG is also being observed in many Pakistani PLHIV [41, 42]. We also observed three unassigned recombinant forms in our cohort, each of which has been observed for the first time in Pakistan, indicating that the Pakistani epidemic is more diverse than previously thought, in terms of subtype composition [43, 44]. Many PWIDs and MSM often exhibit overlapping high-risk behaviors, which increases the possibility of multiple strain acquisition and recombination between strains, allowing the emergence of complex and unique recombinant forms [43–46].

Comparative subtype analysis between the two groups showed a high prevalence of subtype C (14.7%) in the ART-experienced group, which had higher viral loads (Fig 1B). Previous reports have shown that PLHIV infected with subtype C had a higher rate of virological failure than patients infected with other subtypes [47, 48].

Analysis of DRMs in the ART-naïve group showed a high prevalence DRMs conferring resistance against NNRTI and NRTIs. Among NNRTI-associated DRMs, E138A and K103N, and among NNRTI-associated DRMs K219E and K70R were highly prevalent (Table 3). Additionally, a major PI-associated DRM, I84V was observed only in one patient (Table 3). Similarly, DRM analysis of the ART-experienced group showed a high prevalence of DRMs against NNRTI, and NRTIs. Among these, DRMs E138A, K103N, G190A, V179T, M184V, K219E, K70R, and D67N associated with resistance against NNRTI or NRTI were highly prevalent (Table 3). Additionally, in two ART-experienced patients, with high viral loads, the coexistence of DRM M46I and I54V was observed; this combination can cause resistance against all PIs except darunavir/r [27, 49]. Similarly, I47V and V82M/A/S were seen in one patient each, conferring resistance against atazanavir/r, lopinavir/r, and darunavir/r (Table 2).

The DRM E138A has previously been observed as a predominant mutation among ART-experienced Pakistani PLHIV [18]. Similarly, DRM Y115F has also been reported in Pakistani PLHIV [19]. The high prevalence of E138A has also been observed in ART-naïve PLHIV from France (9.3% in 2013) [50], Europe (8.3%), Portugal (13.2%), and North America (15%) [51, 52]. Similarly, DRM M184V has been reported as the most common DRM in India [53, 54]. A DRM analysis from Northern India found 89.8% DRMs in ART failure patients, where DRMs M184V, M41L, D67N, T215Y, and K103N, G190A, Y181C and A98G associated with resistance against reverse transcriptase inhibitors were most prevalent. DRMs against PI were observed in 10.9% of patients, with L10I, V,82A and L89V being the most frequent [55].

Interestingly, we observed a high frequency of certain mutations classified as rare or unusual mutations by the HIV Stanford drug resistance database [27], in both ART-naïve and ART-experienced groups (Table 2). Among these mutations, V179I and K238E were observed in the reverse transcriptase region, while K20I, K20R, V82I, and L10I/V were observed in the protease region in high prevalence. These DRMs against PI were classified as resistance-conferring mutations in the International AIDs society report on DRMs [28]. A study by Lambert *et al*. from France reported a high prevalence of V179I in non-B subtype sequences from ART-naive individuals [56]. Similarly, mutation V179I was also found in rilpivirine-treated samples in vitro [57], where the mutation V179I alone does not reduce susceptibility against rilpivirine but it can increase the rilpivirine resistance in combination with other mutations such as Y181C or L100I+Y181C [57]. Moreover, a clinical trial revealed that V179I mutation was not associated with an increased risk of virological failure [58]. Further studies are needed to determine the effect of these point mutations on drug susceptibility, especially in our part of the world where treatment options are limited and newer-generation NNRTIs are not available.

In our cohort according to the WHO-2009 SDRM list, we observed a high prevalence of certain SDRMs in ART-naïve individuals. SDRMs K103N (NNRTI) and K219E (NRTI) were highly prevalent in the ART-naïve group (Table 2), while SDRM I84V (PI) was also observed in one PLHIV only (Table 2). WHO has classified SDRMs as low (<5%), moderate (5%-15%), and high (>15%) based on geographical distribution [59]. In our cohort, the most frequently found NRTI SDRM was K219E and NNRTI SDRM was K103N (Table 2). SDRM K103N reportedly has the longest mean survival time of about 5.3 years [60]. K103N has also been reported as the predominant SDRM in South Korea (6.1%), China (5%), and Iran (5%) [61–63]. Likewise, SDRM K219E is a thymidine analog mutation (TAM) and reportedly develops significantly more quickly in patients receiving tenofovir (mean of 0.017 mutations per month) than in patients receiving zidovudine (0.006 mutations per month) [64, 65]. Interestingly, the high prevalence of SDRM K219E has not yet been reported in other countries [60]. These SDRMs show a high impact on baseline ART susceptibility. In Pakistan, according to the previous National HIV treatment guidelines, the preferred ART was a combination of zidovudine (AZT), lamivudine (3TC) with efavirenz (EFV) which has since been updated to tenofovir (TDF), and lamivudine (3TC) with efavirenz (EFV) and more recently to tenofovir (TDF) and lamivudine (3TC) with dolutegravir (DTG). However, in special circumstances, zidovudine (AZT), efavirenz (EFV) abacavir (ABC), and lopinavir/ritonavir (LPV/r) may still be used [17, 23]. We observed that most DRMs were directed against RPV (68.5%), ETR (60%), NVP (55.2), and EFV (52.4%) in the NNRTI class, AZT (30%) and ABC (26.5%) in NRTI class; and ATV/r (9%) and LPV/r (8.3%) in PI class (Fig 2). The results suggest that NRTI/NNRTI-based first-line regimen in Pakistan is increasingly becoming ineffective as a result of these DRMs.

In the next stage, we performed genetic variability analysis of the DRM sites. Shannon entropy analysis of the DRM sites identified sites 20, 103, 138, 179, 184, 210, 219, 221, 225, 227, 230, and 238 in the ART-naïve group and sites 20, 103, 138, 179, 184, 190, 219, 221, 230 and 238 in ART-experienced group to have higher than mean entropy (Table 2). Since high Shannon entropy in different regions of the genome correlates with instability [66], the regions with high entropy may be susceptible to further mutations or changes (such as purification of mutations) [66]. This finding is supported by a study from Onsongo et al., where they found high entropy scores for DRM sites in pol sequenced from ART-naive individuals [10]. In the next step, we performed a selection pressure analysis of the DRM sites. Selection pressure is the ratio of synonymous (nucleotide mutations that do not change the amino acid translation) and non-synonymous (nucleotide mutations that change the amino acid translation) mutations called dN/dS [67]. Ratio <1 indicates negative selection pressure, indicating that sites are under constrain, while positive selection (dN/dS ratio is > 1), indicates that amino acid changes are favored i.e., they increase the organism’s fitness and the conditions favor the organism to adapt [67]. In this study, we found DRM sites 82, 184, and 219 to be under positive selection pressure, while the rest of the sites were under negative selection pressure (Table 2). This negative selection and high entropy of most DRM sites indicate that the sites are under genetic constraints either because the replicative fitness of the virus is affected or they are in a transient phase of evolution and will further evolve to a more favorable state [68]. Finally, evolutionary rate analysis revealed that ART-naïve individuals had high mean evolutionary rate than ART-experienced individuals. The higher rate might indicate that the virus in ART-naïve is in the acute phase of infection, and the absence of ART allows the rapid expansion of the viral populations [69]. Furthermore, since most ART-naïve individuals exhibited high DRM prevalence, a high evolution rate might be an effort on the part of the virus to revert to the more stable form, in the absence of ART [69]. This hypothesis can be supported by the high entropy values of most DRM sites in ART-naïve individuals.

We anticipate certain limitations of the study including the small sample size due to the convenience sampling strategy and amplification of only reverse transcriptase and protease genes as none of the participants were on integrase inhibitors. A study on larger samples (from different HIV treatment centers across the country) including data on integrase resistance could shed light on the effectiveness of ART in Pakistan and provide an even more clear picture of the HIV-1 epidemic and drug resistance in Pakistan.

In conclusion, the increasing rates of DRM variants in our cohort are concerning, which eventually can lead to ART failure, particularly the first-line drugs [29, 70]. Further studies are needed for surveillance of drug resistance mutations in newly infected ART naïve patients in Pakistan to encounter the virus more efficiently. This study also warrants the need for comprehensive genetic analysis, surveillance, and control of drug-resistant variants that if become epidemic strains can severely impact HIV treatment modalities as well as control measures.
